# Body Composition in Late Midlife as a Predictor of Accelerated Age-associated Deficit-accumulation From Late Midlife into Old Age: A Longitudinal Birth Cohort Study

**DOI:** 10.1093/gerona/glac233

**Published:** 2022-11-26

**Authors:** Markus J Haapanen, Tuija M Mikkola, Lauri Kortelainen, Juulia Jylhävä, Niko S Wasenius, Eero Kajantie, Johan G Eriksson, Mikaela B von Bonsdorff

**Affiliations:** Folkhälsan Research Center, Helsinki, Finland; Department of Medical Epidemiology and Biostatistics, Karolinska Institutet, Stockholm, Sweden; Folkhälsan Research Center, Helsinki, Finland; Clinicum, Faculty of Medicine, University of Helsinki, Helsinki, Finland; Folkhälsan Research Center, Helsinki, Finland; Department of Health and Social Management, University of Eastern Finland, Kuopio, Finland; Department of Medical Epidemiology and Biostatistics, Karolinska Institutet, Stockholm, Sweden; Faculty of Social Sciences (Health Sciences) and Gerontology Research Center, Tampere University, Tampere, Finland; Folkhälsan Research Center, Helsinki, Finland; Department of General Practice and Primary Health Care, University of Helsinki, Helsinki, Finland; Department of Public Health and Welfare, Population Health Unit, Finnish Institute for Health and Welfare, Helsinki, Finland; PEDEGO Research Unit, Medical Research Center Oulu, Oulu University Hospital, University of Oulu, Oulu, Finland; Folkhälsan Research Center, Helsinki, Finland; Department of Obstetrics and Gynecology and Human Potential Translational Research Programme, Yong Loo Lin School of Medicine, National University Singapore, Singapore; Folkhälsan Research Center, Helsinki, Finland; Gerontology Research Center and Faculty of Sport and Health Sciences, University of Jyväskylä, Jyväskylä, Finland

**Keywords:** Body composition, Frailty, Life-course, Risk factor

## Abstract

**Background:**

Body mass index (BMI) may not be an optimal predictor of frailty as its constituents, lean and fat mass, may have opposite associations with frailty.

**Methods:**

A linear mixed model analysis was performed in the Helsinki Birth Cohort Study (*n* = 2 000) spanning from 57 to 84 years. A 39-item frailty index (FI) was calculated on three occasions over 17 years. Body composition in late midlife included BMI, percent body fat (%BF), waist-to-hip ratio (WHR), lean mass index (LMI), and fat mass index (FMI).

**Results:**

Mean FI levels increased by 0.28%/year among men and by 0.34%/year among women. Among women, per each kg/m^2^ higher BMI and each unit higher %BF the increases in FI levels per year were 0.013 percentage points (PP) steeper (95% CI = 0.004, 0.023) and 0.009 PP steeper (95% CI = 0.002, 0.016) from late midlife into old age. Among men, per each 0.1-unit greater WHR the increase in FI levels was 0.074 PP steeper per year (95% CI = −0.0004, 0.148). Cross-sectionally, greater FMI and LMI in late midlife were associated with higher FI levels but the direction of the association regarding LMI changed after adjustment for FMI. The categories “high FMI and high LMI” and “high FMI and low LMI” showed the highest FI levels relative to the category “low FMI and low LMI”.

**Conclusions:**

In late midlife, greater adiposity (%BF) among women and abdominal obesity (WHR) among men may predispose to higher levels of frailty from late midlife into old age. Greater lean mass alone may be protective of frailty, but not in the presence of high fat mass.

Frailty is a geriatric syndrome characterized by reduced homeostatic reserves and an impaired response to stressors, which predispositions people with frailty at risk of adverse health outcomes (1). The results of a recent meta-analysis show higher waist circumference and  underweight or obese body mass index (BMI) to be associated with an elevated risk of frailty (2). Although a strong predictor of frailty, BMI has been criticized because its different constituents, namely lean and fat mass, may have opposite associations with frailty. Thus, individuals with a similar BMI may present with different body compositions and consequent risk of frailty.

Aging accompanies changes to body composition including increasing fat mass and decreasing lean mass (3). Jointly assessed fat and lean mass should be studied in longitudinal settings to further our understanding of the association between body composition and frailty. Thus far, fat mass indices have not been observed to be associated with prevalent frailty (4). However, fat mass may be associated with increased risk of frailty through higher levels of inflammation (5) and impairment of muscle quality because of higher fat infiltration (6). Some (4) but not all (7) cross-sectional studies show higher lean mass indices associating with a lower prevalence of frailty. In older populations, higher lean mass indices have been associated with better survival (8), providing an alternative to BMI, which predicts survival better when measured earlier in life (9). Omitting the consideration of mutual effects, fat, and lean mass may induce confounding bias given their positive correlativeness (10). Longitudinally, compared to men without low lean mass or obesity, men with obesity alone and sarcopenic obesity (low lean mass and obesity) both had a two-fold increased risk of frailty (11).

In this study, joint assessment of fat and lean mass in late midlife were performed on participants of the Helsinki Birth Cohort Study and their level of frailty tracked over 17 years into old age using a frailty index (FI). We hypothesize higher levels and faster increases in frailty among participants with body compositions characterized with higher fat mass.

## Materials and Methods

### Study Design

**Figure 1.**
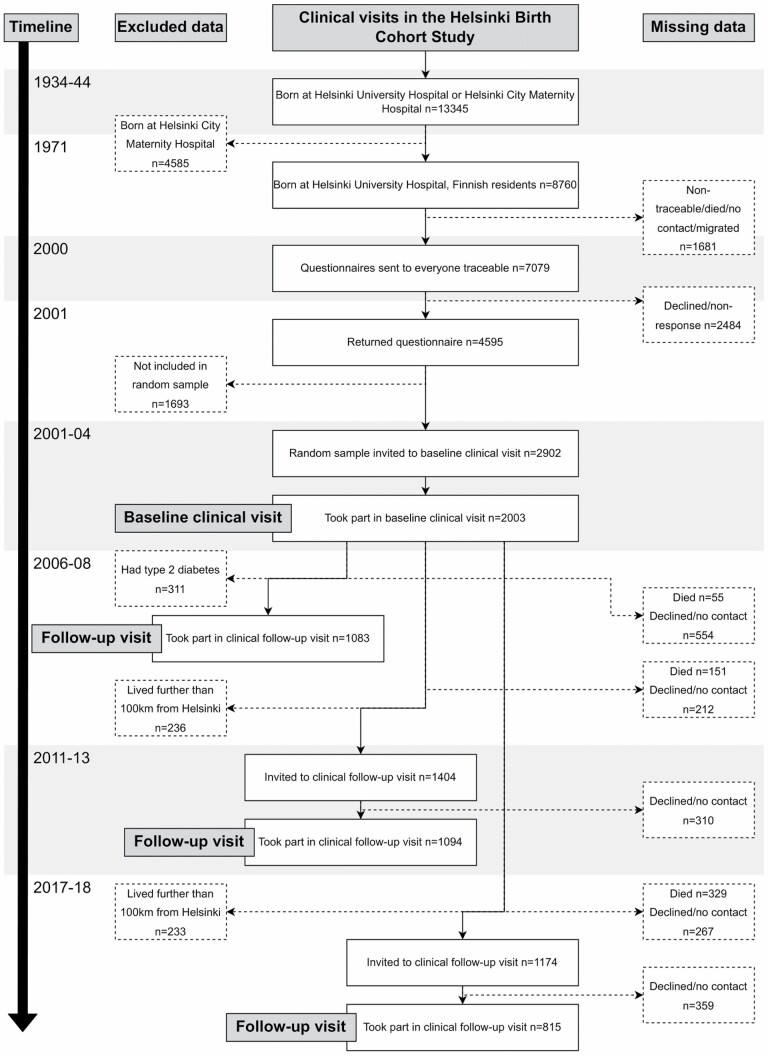
Flowchart of participants in the Helsinki Birth Cohort Study.

Participants of the Helsinki Birth Cohort Study were born at Helsinki University Central Hospital between 1934 and 1944, visited child welfare clinics in the city and lived in Finland in 1971 when a unique personal id number had been allocated to all residents of Finland (12). Figure 1 presents a flowchart over the study population. The present study uses information from the baseline clinical investigation conducted between 2001 and 2004 (*n* = 2 003; mean age = 61.5 years; *SD* = 2.7 years) and clinical follow-up visits conducted in 2011–2013 (*n* = 1 094; mean age = 71.1 years; *SD* = 2.7 years) and 2017–2018 (*n* = 815; mean age = 75.9 years; *SD* = 2.7 years). Body composition and anthropometry were assessed at baseline and a 39-item FI (13) was calculated at all three occasions. The study was approved by the coordinating and Epidemiology and Public Health Ethics Committees of the Hospital District of Helsinki and Uusimaa and that of the National Public Health Institute, Helsinki.

### Frailty Index Assessed Three Times During  2001–2018

The previously published deficit-accumulation based 41-item FI (13) includes diseases, clinical measurements, laboratory test values, functioning measures, and information on general health. We followed the standard procedure (14) in its creation and found its distribution according to age and sex similar to those of published deficit-accumulation based FIs (14–16). For the present study, the deficits *BMI* and *waist-to-hip ratio* were among the studied body composition variables and were excluded from the FI. The resulting 39-item FI was used for our analyses; the 39 items and their scoring into deficits can be found in Supplementary Table 1. The FI could be calculated for 99.6%, 99.9%, and 99.1% of participants participating at each consecutive clinical visit, respectively (13). A total of 2000 participants had data on FI available from at least one clinical measurement occasion.

### Body Composition and Anthropometry Assessed at Clinical Baseline During 2001–2004

Measured weight rounded to the nearest 0.1 kg and height to the nearest 0.1 cm were used in the calculation of BMI, expressed as kg/m². Waist-to-hip ratio (WHR) was calculated by dividing waist circumference with hip circumference. Percentage body fat (%BF) and fat mass were assessed by bioelectrical impedance analysis using the InBody 720 eight-polar tactile electrode system (Biospace Co, Ltd, Seoul, Korea) (17). To calculate lean mass, fat mass was subtracted from total body weight. Fat mass index (FMI) and lean mass index (LMI) were calculated as fat or lean mass in kilograms divided by height in meters to the power of two, expressed as kg/m^2^. FMI and LMI values were then divided into high/low groups according to their median values separately for men (6.29 kg/m^2^ for FMI and 20.77 kg/m^2^ for LMI) and women (9.20 kg/m^2^ for FMI and 17.93 kg/m^2^ for LMI). This information was used in creating four body composition groups: (i) high FMI and high LMI, (ii) high FMI and low LMI, (iii) low FMI and low LMI, and (iv) low FMI and high LMI, chosen as reference group in our analyses.

### Clinical Covariate Data Obtained During 2001–2004

Self-administered questionnaires were used to assess smoking, coded into never, former, and current smoker. The highest attained occupational status at 5-year intervals between 1970 and 1995 was used to indicate socioeconomic status (SES) in adulthood and was obtained from Statistics Finland (18).

### Statistical Methods

We used linear mixed models to examine the associations between body composition and FI levels at age 57 years and the rate of change in FI levels from late midlife into old age. Age was used as the underlying time scale and centered at 57 years, the youngest age in the data. Other continuous variables were centered at their mean values. We stratified our analyses by sex because of marked differences in body composition between men and women (19) and previously observed sex differences in the association between body composition and frailty (4,20). Body composition and anthropometry were first examined in separate models. FMI and LMI were then studied jointly; first through mutual adjustment of FMI and LMI and then as FMI–LMI categories. We adjusted our models with key lifestyle factors (smoking) that were not already included among the studied variables or in the FI, socioeconomic factors (adult SES), and their interactions with age. To account for sample attrition during the study, we repeated our main analysis assuming missing not at random sample attrition (21). To improve the interpretability of our model estimates, we multiplied the FI by 100 and treat them as a percentage. Estimates of the FI level correspond to percentage (%) lower/higher levels of frailty whereas estimates of the rate of change in FI levels correspond to percentage point (PP) differences of change per year from late midlife into old age. Negative estimates indicate lower levels of frailty at age 57 years or slower increase in FI levels from late midlife into old age. Parametric bootstrap was used to calculate 95 % confidence intervals in the figures. *p*-value < .05 was used to indicate statistical significance. The analyses were performed using the R software (22) packages lme (23) and lmerTest (24).

## Results

### Body Composition Variables Defined as BMI, WHR, %BF, FMI, LMI, and FMI–LMI Categories

Bivariate correlations between body composition variables are shown in Supplementary Table 2. Table 1 shows the cohort characteristics by sex. While mean BMI was similar among men and women (27.5 and 27.7 kg/m^2^), both sexes showed distinct body compositions. Mean WHR (1.0 vs. 0.9) and LMI (20.8 vs.  18.0 kg/m^2^) were higher among men, whereas women showed body compositions characterized by higher mean FMI (9.7 vs. 6.7 kg/m^2^) and %BF (33.9 vs. 23.8 %). FMI – LMI categories identified distinct body composition groups, shown in Supplementary Table 3. As not all participated in the follow-up visit, we tested body composition variables for differences among invited, lost, and dead participants at the follow-up, showing no evidence of body composition affecting participation status (Supplementary Table 4).

**Table 1.** Characteristics of the 2003 Individuals Participating in Baseline Clinical Measurements During 2001–2004

	Total study population (*n* = 2 003)	Women (*n* = 1 075)	Men (*n* = 928)	* p*
	Mean (SD)	Mean (SD)	Mean (SD)	
Participant characteristics assessed at baseline				
Age (years)	61.5 (2.9)	61.5 (2.8)	61.5 (3.0)	.694
Smoking status				<.001
Never smoker, *n*(%)	839 (42.2)	593 (55.7)	246 (26.7)	
Quit smoking, *n*(%)	673 (33.9)	251 (23.6)	422 (45.8)	
Current smoker, *n*(%)	475 (23.9)	221 (20.7)	254 (27.5)	
Body composition				
BMI (kg/m^2^)	27.6 (4.7)	27.7 (5.0)	27.5 (4.2)	.783
Waist-to-hip ratio	0.9 (0.1)	0.9 (0.1)	1.0 (0.1)	<.001
Lean mass index (kg/m^2^)	19.3 (2.3)	18.0 (1.7)	20.8 (1.9)	<.001
Fat mass index (kg/m^2^)	8.3 (3.6)	9.7 (3.7)	6.7 (2.8)	<.001
Percent body fat (%)	29.2 (8.2)	33.9 (6.9)	23.8 (6.0)	<.001
Categories of fat and lean body mass index				.007
High FMI and high LMI, *n*(%)	688 (35.9)	387 (37.5)	301 (34.0)	
High FMI and low LMI, *n*(%)	271 (14.1)	129 (12.5)	142 (16.0)	
Low FMI and high LMI, *n*(%)	270 (14.1)	128 (12.4)	142 (16.0)	
Low FMI and low LMI, *n*(%)	689 (35.9)	388 (37.6)	301 (34.0)	
Adult socioeconomic status				<.001
Manual worker, *n*(%)	671 (33.5)	276 (25.7)	395 (42.6)	
Self-employed, *n*(%)	187 (9.3)	91 (8.5)	96 (10.3)	
Lower official, *n*(%)	858 (42.9)	603 (56.2)	255 (27.5)	
Upper official, *n*(%)	286 (14.3)	104 (9.7)	182 (19.6)	
Frailty index				
Baseline measurement occasion in 2001–2004^a^	0.20 (0.10)	0.21 (0.10)	0.20 (0.10)	.120
Frail (FI≥ 0.25) at baseline, *n*(%)	575 (28.71)	336 (31.26)	239 (25.75)	.007
Follow-up visit in 2011–2013^b^	0.21 (0.10)	0.23 (0.10)	0.19 (0.10)	<.001
Follow-up visit in 2017–2018^c^	0.23 (0.11)	0.24 (0.11)	0.21 (0.10)	<.001

*Notes:* BMI = body mass index; FMI = fat mass index; LMI = lean mass index; SD = standard deviation.

^a^ *n* = 1 995.

^b^ *n* = 1 081.

^c^ *n* = 806.

### Body Composition Variables and the FI Level Assessed in Late Midlife

At the age of 57 years, the mean FI level was 0.161 among men and 0.174 among women. Greater BMI, WHR, %BF, LMI, and FMI were all associated with a higher FI level in late midlife among both men and women after adjustment for smoking and adult SES (Table 2). For each kg/m^2^ unit higher in BMI the percentage increase in FI levels was of similar magnitude for men (0.72%,  *p* < .001) and women (0.63%, *p* < .001). FMI was driving the association among men: per each kg/m^2^ unit higher in FMI the level FI was 1.30% greater among men and 0.92% greater among women (*p*-values < .001). Further adjustment for LMI strengthened the associations. Among women, the association was driven by LMI: per each kg/m^2^ unit higher in LMI the FI level was 1.23% greater among women and 0.77% greater among men (*p*-values < .001). Further adjustment with FMI changed the direction of the associations: per each kg/m^2^ unit higher in LMI the FI level was 0.63% lower among men (*p* = .009) and 0.46% lower among women (*p* = .116). The associations were attenuated when LMI was adjusted with percent body fat (Table 2).

**Table 2.** Body Composition Variables and their Associations with Point Estimates of the FI Level at Age 57 Years and the Annual Rate of Change in FI Levels from Late Midlife into Old Age

	Level^a^	95 % CI	* p*	Rate of change^b^	95 % CI	* p*
Body composition variable^c^						
BMI (kg/m^2^)						
Women	0.63	0.50, 0.76	<.001	0.013	0.004, 0.023	.007
Men	0.72	0.55, 0.88	<.001	0.008	−0.004, 0.021	.216
Waist-to-hip ratio						
Women	3.98	3.08, 4.89	<.001	0.009	−0.052, 0.070	.766
Men	4.80	3.71, 5.89	<.001	0.074	−0.0004, 0.148	.051
Percent body fat (%)						
Women	0.48	0.38, 0.58	<.001	0.009	0.002, 0.016	.012
Men	0.61	0.49, 0.73	<.001	0.001	−0.007, 0.010	.739
Lean mass index (kg/m^2^)						
Women	1.23	0.81, 1.64	<.001	0.035	0.007, 0.063	.016
Men	0.77	0.38, 1.16	<.001	0.010	−0.018, 0.038	.493
Women^d^	−0.46	−1.03, 0.11	.116	0.023	−0.015, 0.062	.236
Men^d^	−0.63	−1.11, −0.16	.009	0.019	−0.014, 0.053	.262
Women^e^	0.25	−0.22, 0.72	.303	0.025	−0.007, 0.057	.128
Men^e^	−0.03	−0.45, 0.38	.871	0.014	−0.016, 0.045	.353
Fat mass index (kg/m^2^)						
Women	0.93	0.74, 1.11	<.001	0.018	0.005, 0.031	.008
Men	1.29	1.04, 1.54	<.001	0.008	−0.012, 0.027	.440
Women^f^	1.08	0.81, 1.34	<.001	0.010	−0.008, 0.029	.274
Men^f^	1.55	1.23, 1.86	<.001	-0.001	−0.025, 0.024	.979

*Notes:* BMI = body mass index; CI = confidence interval; FI = frailty index;.

^a^In FI × 100 units, which correspond percentage increases/decreases in FI levels at age 57 years (mean FI level at age 57 years was 0.161 among men and 0.174 among women).

^b^In percentage points per year from late midlife into old age (mean annual rate of change in FI levels from late midlife into old age was 0.28 percent/year among men and 0.34 percent/year among women). Point estimates are derived from models with the age × body composition interaction.

^c^Analyzed individually. Linear mixed model adjusted with age, smoking, and adult socioeconomic status.

^d^Adjusted additionally for fat mass index (kg/m^2^).

^e^Adjusted additionally for percent body fat (%).

^f^Adjusted additionally for lean mass index (kg/m^2^).

FMI–LMI categories characterized by “high FMI” were associated with a higher FI level in late midlife in both sexes combined (Supplementary Table 5). Relative to the category “low FMI and high LMI”, membership in the categories “high FMI and high LMI”, “high FMI and low LMI” and “low FMI and low LMI” were associated with 6.90%, 4.54% (*p*-values < .001) and 1.27% (*p* = .112) greater FI levels in late midlife, respectively.

### Body Composition Variables and the Rate of Change in FI Levels from Late Midlife into Old Age

**Figure 2.**
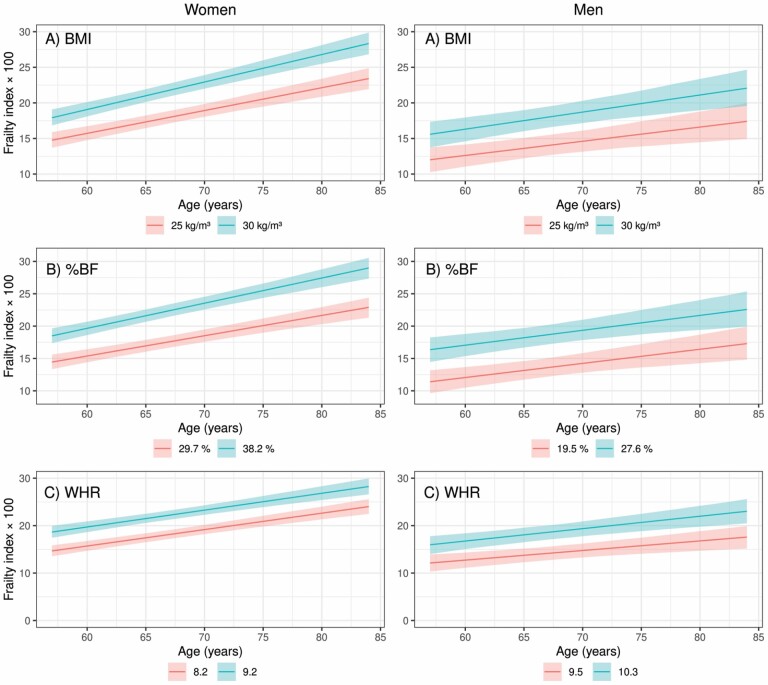
(**A**–**C**) Mean frailty index levels (FI × 100) as a function of age from late midlife into old age in the Helsinki Birth Cohort Study for body composition variables which were significantly associated with the rate of change in FI levels from late midlife into old age: shown (**A**) separately for men and women in groups of BMI (<25.0 kg/m^2^, ≥25.0 and ≤30.0 kg/m^2^, >30.0 kg/m^2^), (**B**) separately for men and women in groups of percent body fat (%BF) (sex-specific 25th and 75th percentile), (**C**) separately for men and women in groups of waist-to-hip ratio (WHR) (sex-specific 25th and 75th percentile). Adjusted with smoking, adult socioeconomic status, and their interactions with age. Parametric bootstrap was used to calculate 95% confidence intervals.

The mean increase in FI levels was 0.28%/year among men and 0.34%/year among women from late midlife into old age. Greater BMI, %BF, LMI, and FMI were associated with a steeper increase in FI levels from late midlife into old age among women when adjusted for smoking and adult SES (Table 2). Among women, per each  kg/m^2^ higher in BMI and per each unit higher in %BF the respective increases in FI levels were 0.013 PP (95% CI = 0.004, 0.023) and 0.009 PP (95% CI = 0.002, 0.016) steeper per year from late midlife into old age. Figure 2A–C present the development of FI levels in groups of BMI (cut-offs at 25 kg/m^2^ and 30 kg/m^2^), %BF, and WHR (cut-offs at sex-specific 25th and 75th percentiles). Each kg/m^2^ higher FMI and LMI at baseline were associated with a steeper increase in FI levels from late midlife into old age. However, adjustment of FMI with LMI, and vice versa, attenuated these associations. Among men, per each 0.1-unit greater WHR the increase in FI levels was 0.074 PP steeper per year (95% CI = −0.0004, 0.148) from late midlife into old age. Estimates of Table 2-predictors assuming missing not at random sample attrition showed parallel results (Supplementary Table 6).

**Figure 3.**
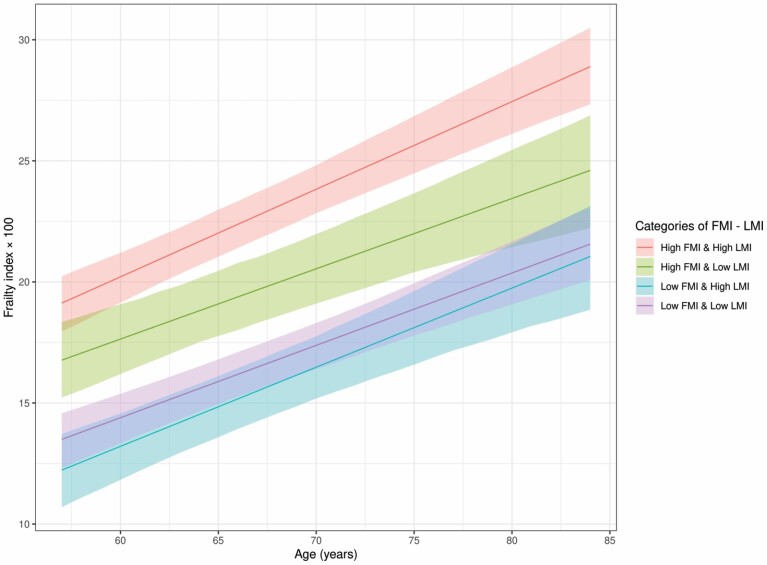
Mean frailty index levels (FI × 100) as a function of age from late midlife into old age in the Helsinki Birth Cohort Study shown in FMI–LMI categories. The cut-off for low/high FMI was 6.29 kg/m^2^ for men and  9.20 kg/m^2^ for women and the cut-off for low/high LMI was 20.77 kg/m^2^ for men and 17.93 kg/m^2^ for women. Adjusted with smoking, adult socioeconomic status, and their interactions with age. Parametric bootstrap was used to calculate 95% confidence intervals.

Figure 3 presents the development of FI levels in FMI–LMI categories and show that the FI level in late midlife was the highest in the category “high FMI and high LMI”, followed by “high FMI and low LMI”, which persisted from late midlife into old age. The lowest levels of frailty in late midlife were observed in the groups where FMI was low. However, we observed no associations between FMI–LMI categories and the rate of change in FI levels from late midlife into old age (Supplementary Tables 5 and 7).

## Discussion

Longitudinal studies show that higher BMI is associated with an increased risk of frailty. However, BMI is not an optimal predictor of health outcomes as its different constituents, namely lean and fat mass, may have opposite associations with frailty. In this study of community-dwelling individuals, the FI levels of women with a higher BMI and greater adiposity (%BF) and men with greater abdominal obesity (WHR) in late midlife increased steeper from late midlife into old age (ages 57–84 years). Cross-sectionally, fatter body composition in late midlife was strongly associated with a higher level of frailty among both sexes. Higher LMI seemed to be protective of frailty particularly among men, but no longer in the presence of high FMI. Our findings suggest potential improvement to frailty risk prevention through identification of high BMI and %BF among women and WHR among men in late midlife. Joint assessment of body composition variables may be of significance, as we observed the direction of the association between LMI and frailty level change after adjustment with FMI.

### Cross-sectional Findings

In line with previous studies, we observed positive cross-sectional associations between higher BMI (25) and %BF (26,27) associating with higher FI levels. Fewer previous studies have focused on indices of body composition relative to height including FMI (4), LMI (4,28), skeletal muscle index (7), and waist-to-height ratio (20). In line with observations in the present study, Soh et al. (4) observed higher LMI associating with a lower prevalence of frailty among older community-dwelling Koreans. However, the authors observed no associations between FMI or %BF and frailty. Williams et al. (7) found that muscle density, rather than skeletal muscle index, was associated with frailty among patients with cancer. Kim et al. (20) suggested mediating effects of waist-to-height ratio on the association between obesity and frailty among older Korean women. In their study, the risk attributable to obesity was attenuated after adjustment for waist-to-height ratio. Taken together, the evidence from individually analyzed indices of fat and lean mass and frailty is inconsistent but suggests potentially higher risk in individuals with high indices of obesity and potential protection from risk through higher lean indices.

We were able to extend these findings through joint assessment of FMI and LMI and show that FMI seems to drive the association between higher BMI and FI levels. Higher LMI may be protective of frailty among men, but not in the simultaneous presence high FMI. This dominating effect of fat indices was further supported in FMI–LMI categories, where the level of frailty was the highest in groups where FMI was high and lowest where LMI was low. Our sample, consisting of relatively fit older adults, was characterized with higher LMI and average FMI values when compared with reference values from a European cohort (19). Our results highlight that FMI and LMI act together, and optimally, both should be accounted for in future studies assessing body composition.

### Longitudinal Findings

In the present study, we observed no associations between jointly assessed FMI–LMI categories and the rate of change in FI levels from late midlife into old age. In other words, the pace at which the participants’ level of frailty increased per year was similar in jointly assessed FMI–LMI categories. However, those with higher fat mass indices in late midlife were frailer and stayed so until old age compared with those with lower fat mass indices. The participants who were most frail throughout were those with high fat and lean mass indices. In the previous study by Hirani et al. (11) the risk of frailty was minimally incremented when measures of high adiposity were combined with low lean mass. In their study of men aged 70 years and older, men who had either low muscle mass or sarcopenic obesity (combined low muscle mass and high fat percentage) both had an approximately two-fold risk of frailty during the study period compared with men without obesity or low muscle mass. In this study of initially younger men and women, it was not low muscle mass that was associated with participants becoming frailer earlier, but instead indices of higher body mass (BMI) and adiposity (%BF) among women and abdominal obesity (WHR) among men. This means that among men and women, there was evidence of an interaction between sex and body composition on the participants’ annual pace of increase in frailty levels from midlife into old age. Our results agree with previous observations of higher BMI associating with incident frailty (29), its progression (29,30), and of higher WHR associating with the progression of frailty (29,30) and extend them by providing evidence of differences in body composition between men and women that increase the risk of becoming frailer earlier.

Furthermore, we found that BMI assessed in late midlife was not consistently associated with the risk of frailty. It was associated with risk among women, who also have higher levels of total body fat than men in given BMI values (31). This means that BMI better captures fatness among women than men. The potential protective association of higher LMI and lower risk of frailty (4) may conflate the use of BMI in estimating the risk of frailty among men. Our results suggest that WHR may outperform BMI among men in capturing fatness and their consequent risk of frailty.

### Mechanisms

Aging involves changes to body composition including increasing fat mass, decreasing lean mass, and an altering distribution of lean/fat mass (3). Higher adiposity in particular has been associated with frailty in a life-course perspective, where abnormal patterns of childhood growth (32), midlife obesity (33,34), weight gain (35), and long-term obesity (36) were all associated with an increased risk of frailty. Higher levels of adiposity may contribute to a chronic pro-inflammatory state (5) and infiltration of lipids to other tissues including muscle (6). Insulin resistance (37,38) and other cardiovascular risk factors (39) may follow, which have been shown to increase the risk of frailty (34,40). Besides cardiovascular risk factors, obesity may also predispose to declining mobility and higher levels of disability (41).

### Strengths and Limitations

Strengths of the present study include detailed clinical data with follow-up from late midlife into old age. We were able to study lean and fat mass indices separately and jointly to further our understanding on the association between body composition and frailty. The FI (13) used in the study emphasizes weight loss less (one of the 39 deficits) than the frailty phenotype (one of five criteria), which may help to reduce confounding between body composition and frailty. The study results should be interpreted considering the following weaknesses. First, body composition was assessed using bioelectric impedance analysis, which is subject to inferior validity (42) and may overestimate lean and underestimate fat mass relative to those assessed by dual-energy X-ray absorptiometry (43). Second, while the FI used in the study had exceeded the minimum of 30 deficits, it does not include deficits related to cognitive test results or sensory problems due to insufficient data. While two deficits were excluded for the present study, the FI has not been shown to be sensitive to missing deficits (44). Third, moderate sample attrition occurred over the 17-year follow-up. However, we found baseline body composition to be minimally associated with participant status at the follow-up visit. Furthermore, assuming not missing at random sample attrition did not significantly alter the results. Fourth, we used information on body composition at baseline only and we cannot exclude that body composition varied with time in the cohort. Finally, we report findings among relatively fit community-dwelling Caucasians and suggest caution in generalizing the results to other populations or ethnicities.

### Implications

Fat and lean mass showed opposite associations with the risk of frailty; we found evidence that higher fat mass overruled lean mass as a predictor of frailty in late midlife. While higher lean mass has previously been suggested to prove beneficial for frailty, its significance may be greatly reduced with high levels of fat mass. There was an interaction between age and waist-to-hip ratio on frailty among men, which meant that men with higher waist-to-hip ratios became frailer earlier. Therefore, avoiding high levels of abdominal obesity in midlife among men could help lowering one’s future risk of frailty. The body composition predisposing women to becoming frailer earlier was different; women who had a higher BMI and %BF became frailer earlier. Avoiding a high BMI and high levels of adiposity (%BF) among women in midlife may help in the prevention of future frailty. The use of multiple/jointly assessed indices of body composition may be useful, as single measures alone may produce inconsistent risk estimates or overestimate risk. Future studies may choose to address, besides fat and lean indices, indices of muscle quality.

## Conclusion

Fat and lean mass showed opposite associations with the risk of frailty in late midlife. Higher fat mass overruled possible protective effects of lean mass on the risk of frailty in late midlife. The FI levels of women with a greater BMI and level of adiposity (%BF) and men with greater abdominal obesity (WHR) increased steeper in the period ranging from late midlife into old age. Avoiding high levels of adiposity/abdominal obesity in midlife may help to reduce one’s future risk of frailty.

## Supplementary Material

glac233_suppl_Supplementary_Material
