# Supplementary material for: Body Composition in Late Midlife as a Predictor of Accelerated Age-associated Deficit-accumulation From Late Midlife into Old Age: A Longitudinal Birth Cohort Study
Source: J Gerontol A Biol Sci Med Sci. 2022 Nov 26;78(6):980–7. doi: 10.1093/gerona/glac233 (PMC10235203; doi:10.1093/gerona/glac233)
Supplement: glac233_suppl_Supplementary_Material [file glac233_suppl_supplementary_material.pdf]

Supplementary Material

Body composition predicting age-associated deficit accumulation across 17 years from late midlife into old age

Supplementary Table 1. List of the 39 variables included in the frailty index in the Helsinki Birth Cohort Study.

Supplementary Table 2. Bivariate correlations between body compositions variables according to sex.

Supplementary Table 3. Body composition at clinical baseline according to sex-specific median groups of fat and lean mass index.

Supplementary Table 4. Characteristics of participants participating in baseline clinical examinations according to participant status at the follow-up visit.

Supplementary Table 5. Categories of fat and lean mass index predicting point estimates of the FI level at age 57 years and the rate of change in FI levels from late midlife into old age.

Supplementary Table 6. Body composition variables and their associations with point estimates of the FI level at age 57 years and the annual rate of change in FI levels from late midlife into old age assuming missing not at random sample attrition.

Supplementary Table 7. Categories of fat and lean mass index predicting point estimates of the FI level at age 57 years and the rate of change in FI levels from late midlife into old age assuming missing not at random sample attrition.

Supplementary Table 1. List of the 39 variables included in the frailty index in the Helsinki Birth Cohort Study.

	Variable name	Scoring
1	Self-reported angina pectoris as diagnosed by a doctor[a]	Yes=1; no=0
2	Self-reported asthma as diagnosed by a doctor[a], special reimbursement for obstructive airway disease medication[b]	Yes=1; no=0
3	Self-reported cancer as diagnosed by a doctor[a]	Yes=1; no=0
4	Self-reported claudication as diagnosed by a doctor[a]	Yes=1; no=0
5	Special reimbursement for antiarrhythmic medication[b]	Yes=1; no=0
6	Self-reported depression by a doctor[a]	Yes=1; no=0
7	Self-reported diabetes as diagnosed by a doctor[a], special reimbursement for diabetes medication[b]	Yes=1; no=0
8	Self-reported emphysema as diagnosed by a doctor[a]	Yes=1; no=0
9	Special reimbursement for glaucoma medication[b]	Yes=1; no=0
10	Self-reported heart failure as diagnosed by a doctor[a]	Yes=1; no=0
11	Self-reported hypertension as diagnosed by a doctor[a], special reimbursement for antihypertensive drugs[b]	Yes=1; no=0
12	Self-reported myocardial infarct as diagnosed by a doctor[a]	Yes=1; no=0
13	Self-reported osteoporosis as diagnosed by a doctor[a]	Yes=1; no=0
14	Self-reported stroke as diagnosed by a doctor[a]	Yes=1; no=0
15	General health[c]	Poor=1; fair=0.75; good=0.50; very good=0.25; excellent=0
16	Health compared to one year ago[c]	Worse=1; same/better=0
17	Health limits vigorous activities[c]	Yes=1; yes a bit=0.50; no=0
18	Health limits moderate activities[c]	Yes=1; yes a bit=0.50; no=0
19	Health limits lifting or carrying groceries[c]	Yes=1; yes a bit=0.50; no=0
20	Health limits climbing several flights of stairs[c]	Yes=1; yes a bit=0.50; no=0
21	Health limits bending, kneeling, or stooping[c]	Yes=1; yes a bit=0.50; no=0
22	Health limits walking more than a kilometre[c]	Yes=1; yes a bit=0.50; no=0
23	Health limits walking more than 100 metres[c]	Yes=1; yes a bit=0.50; no=0
24	Health limits bathing or dressing[c]	Yes=1; yes a bit=0.50; no=0
25	Physical health limits the kind of work or other activities[c]	Yes=1; no=0
26	Bodily pain[c]	Severe/very severe=1; moderate=0.75; mild=0.50; very mild=0.25; no pain=0
27	Feel full of life[c]	None=1; some/little=0.50; all/most/good bit=0
28	Feel tired[c]	All/most/good bit=1; some/little=0.50; none=0
29	Heart rate (bpm)	<60 or >100=1; ≥ 60 and ≤ 100=0
30	Systolic blood pressure measured to be $\geq$ 160 mmHg or diastolic blood pressure measured to be $\geq$ 100 mmHg	Yes=1; no=0
31	Abnormal fasting glucose ($\geq$ 6.1 mmol/l)[d]	Yes=1; no=0
32	High total cholesterol level ($>$ 5.0 mmol/l)[d]	Yes=1; no=0

33	Low high-density lipoprotein level (men < 1.00 mmol/l, women < 1.20 mmol/l)[d]	Yes=1; no=0
34	Abnormal alanine amino transferase / aspartate transaminase level (ALT > 50 U/l for men and > 35 U/l for women; AST > 45 U/l for men and > 35 U/l for women)[d]	Yes=1; no=0
35	Weekly metabolic equivalent of physical activity in hours (METh/week)	Lowest quintile stratified by sex=1; other=0
36	Less interested in other people than before[e]	Yes=1; no=0
37	Changes in sleeping pattern[e]	Yes=1; more tired in the morning=0.50; sleep as well as before=0
38	Changes in appetite[e]	Less than usual=1; no change=0
39	Weight loss[e]	2.5 kg or greater=1; weight has been stable=0

Note. [a]Assessed using questionnaires at clinical baseline and follow-up visits; [b]prescription medicines reimbursed out of National Health Insurance until the year 2017; [c]from the RAND-36/SF-36 questionnaire (1); [d]cut-offs indicating abnormal test results; [e]from the Beck Depression Inventory (BDI) (2) questionnaire.

1. RAND. The RAND 36-Item Health Survey The RAND 36-Item Health Survey. *Heal (San Fr*. 1992;2(March):91-97. doi:10.1002/hec.4730020305.
2. Beck AT, Steer RA BG. *Manual for the Beck Depression Inventory-II*. San Antonio TX: Psychological Corporation; 1996.

Supplementary Table 2. Bivariate correlations between body compositions variables according to sex.

Men						
	BMI	LMI	FMI	WHR	%BF	Legend
BMI	1					<0.5
LMI	0.8472	1				-0.6
FMI	0.9344	0.6024	1			-0.7
WHR	0.6630	0.4463	0.6905	1		-0.8
%BF	0.8121	0.4139	0.9427	0.6955	1	-0.9
						>0.9

Women					
	BMI	LMI	FMI	WHR	%BF
BMI	1				
LMI	0.8602	1			
FMI	0.9721	0.7167	1		
WHR	0.5360	0.4887	0.5088	1	
%BF	0.8757	0.5421	0.9484	0.4947	1

Note. Abbreviations: BMI=body mass index; LMI=lean mass index; FMI=fat mass index; WHR=waist to hip ratio; %BF=percent body fat. P-values for all bivariate correlations < 0.001.

Supplementary Table 3. Body composition at clinical baseline according to sex-specific median groups of fat mass index and lean mass index.

	Fat mass index low[a]		Fat mass index high[a]		p
	Lean mass index low[b]	Lean mass index high[b]	Lean mass index low[b]	Lean mass index high[b]	
	N=689	N=270	N=271	N=688	
Age in years, mean (SD)	61.4 (2.9)	61.0 (2.5)	62.1 (3.3)	61.6 (2.9)	0.015
Women, n (%)	388 (56.3)	128 (47.4)	129 (47.6)	387 (56.3)	0.007
Height (cm), mean (SD)	169.6 (8.9)	172.4 (8.4)	167.8 (8.6)	168.9 (9.2)	<0.001
Weight (kg), mean (SD)	67.9 (9.5)	78.9 (9.6)	77.6 (8.4)	92.2 (14.4)	<0.001
BMI (kg/m^2), mean (SD)	23.5 (1.9)	26.4 (1.4)	27.5 (1.4)	32.3 (4.1)	<0.001
Waist-to-hip ratio, mean (SD)	0.9 (0.1)	0.9 (0.1)	0.9 (0.1)	1.0 (0.1)	<0.001
Body fat mass (kg), mean (SD)	16.5 (4.3)	18.2 (4.0)	25.0 (4.0)	32.0 (9.3)	<0.001
Percent body fat, mean (SD)	24.6 (6.3)	23.4 (5.7)	32.7 (5.6)	34.8 (7.3)	<0.001
Body lean mass (kg), mean (SD)	51.4 (9.3)	60.7 (10.2)	52.5 (8.5)	60.2 (11.2)	<0.001
Fat mass index (kg/m^2), mean (SD)	5.8 (1.7)	6.2 (1.6)	9.0 (1.8)	11.4 (3.7)	<0.001
Lean mass index (kg/m^2), mean (SD)	17.7 (1.6)	20.3 (1.7)	18.5 (1.4)	20.9 (2.0)	<0.001
Smoking status					0.491
Never smoker, n (%)	305 (44.5)	113 (42.3)	101 (37.5)	282 (41.5)	
Quit smoking, n (%)	218 (31.8)	87 (32.6)	103 (38.3)	239 (35.1)	
Current smoker, n (%)	163 (23.8)	67 (25.1)	65 (24.2)	159 (23.4)	
Adult SES					<0.001
Manual worker, n (%)	196 (28.4)	84 (31.1)	102 (37.6)	259 (37.6)	
Self-employed, n (%)	54 (7.8)	27 (10.0)	32 (11.8)	69 (10.0)	
Lower official, n (%)	314 (45.6)	117 (43.3)	103 (38.0)	292 (42.4)	
Upper official, n (%)	125 (18.1)	42 (15.6)	34 (12.5)	68 (9.9	
Frailty index					
Baseline measurement in 2001-2004	0.16 (0.09)	0.16 (0.08)	0.21 (0.09)	0.25 (0.10)	<0.001
Follow-up in 2011-2013	0.18 (0.09)	0.18 (0.08)	0.21 (0.09)	0.26 (0.10)	<0.001
Follow-up in 2017-2018	0.20 (0.10)	0.20 (0.09)	0.23 (0.10)	0.28 (0.11)	<0.001

Note. SD=standard deviation; BMI=body mass index; SES=socioeconomic status.

[a]Cut-offs for fat mass index: 6.29 kg/m^2 for men and 9.20 kg/m^2 for women.

[b]Cut-offs for lean mass index: 20.77 kg/m^2 for men and 17.93 kg/m^2 for women.

Supplementary Table 4. Characteristics of participants participating in baseline clinical examinations according to participant status at the follow-up visit.

	**Invited to clinical follow-up visit**	**Declined/no contact/lived further away**	**Died**	**P**
	n=1404	n=448	n=151	
	Mean (SD)	Mean (SD)	Mean (SD)	
**Participant characteristics assessed at baseline**				
Age (years)	61.4 (2.9)	61.7 (3.0)	62.1 (3.1)	0.023
**Smoking status**				<0.001
Never smoker, N (%)	613 (44.0)	182 (41.0)	44 (29.3)	
Quit smoking, N (%)	474 (34.0)	154 (34.7)	45 (30.0)	
Current smoker, N (%)	306 (22.0)	108 (24.3)	61 (40.7)	
**Body composition**				
BMI (kg/m^2)	27.6 (4.5)	27.9 (5.2)	27.6 (4.8)	0.666
Waist-to-hip ratio	0.9 (0.1)	0.9 (0.1)	1.0 (0.1)	<0.001
Lean mass index (kg/m^2)	19.3 (2.2)	19.3 (2.4)	19.5 (2.2)	0.370
Fat mass index (kg/m^2)	8.3 (3.5)	8.6 (4.0)	8.0 (3.6)	0.355
Percent body fat (%)	29.2 (8.1)	29.7 (8.5)	28.2 (8.4)	0.199
**Categories of fat and lean body mass index**				0.057
High FMI & high LMI, N (%)	485 (35.8)	154 (36.4)	49 (34.5)	
High FMI & low LMI, N (%)	169 (12.5)	73 (17.3)	29 (20.4)	
Low FMI & high LMI, N (%)	198 (14.6)	55 (13.0)	17 (12.0)	
Low FMI & low LMI, N (%)	501 (37.0)	141 (33.3)	47 (33.1)	

Note. SD=standard deviation; BMI=body mass index; FMI=fat mass index; LMI=lean mass index.

Supplementary Table 5. Categories of fat and lean mass index predicting point estimates of the FI level at age 57 years and the rate of change in FI levels from late midlife into old age.

	**Level** [a]	**95 % CI**	**P**	**Rate of change** [b]	**95 % CI**	**P**
**Categories of fat and lean mass index** [c]						
High FMI & high LMI	6.90	5.33, 8.46	<0.001	0.035	-0.064, 0.134	0.489
High FMI & low LMI	4.54	2.64, 6.45	<0.001	-0.036	-0.162, 0.089	0.569
Low FMI & low LMI	1.27	-0.29, 2.83	0.112	-0.028	-0.125, 0.069	0.572
Low FMI & high LMI	Ref.			Ref.		

Note. FI=frailty index; CI=confidence interval; FMI=fat mass index; LMI=lean mass index.

[a] In FI × 100 units, which correspond percentage increases/decreases in FI levels at age 57 years (mean FI level at age 57 years was 0.186).

[b] In percentage points per year from late midlife into old age (mean annual rate of change in FI levels from late midlife into old age was 0.34 percent/year). Point estimates correspond to age × body composition variable interactions.

[c] Linear mixed model adjusted with age, smoking, and adult socioeconomic status.

Supplementary Table 6. Body composition variables and their associations with point estimates of the FI level at age 57 years and the annual rate of change in FI levels from late midlife into old age assuming missing not at random sample attrition.

	**Level[a]**	**95 % CI**	**P**	**Rate of change[b]**	**95 % CI**	**P**
**Body composition variable[c]**						
BMI (kg/m^2)						
Women	0.63	0.52, 0.74	<0.001	0.014	0.007, 0.022	<0.001
Men	0.71	0.56, 0.86	<0.001	0.010	-0.001, 0.020	0.080
Waist-to-hip ratio						
Women	3.96	3.25, 4.71	<0.001	0.013	-0.036, 0.065	0.640
Men	4.78	3.84, 5.76	<0.001	0.085	0.026, 0.142	0.004
Percent body fat (%)						
Women	0.48	0.39, 0.56	<0.001	0.009	0.004, 0.015	<0.001
Men	0.60	0.50, 0.71	<0.001	0.003	-0.004, 0.010	0.363
Lean mass index (kg/m^2)						
Women	1.24	0.89, 1.59	<0.001	0.034	0.011, 0.057	0.003
Men	0.80	0.46, 1.15	<0.001	0.007	-0.016, 0.029	0.506
Fat mass index (kg/m^2)						
Women	0.93	0.77, 1.09	<0.001	0.018	0.006, 0.030	<0.001
Men	1.28	1.06, 1.50	<0.001	0.011	-0.005, 0.028	0.188

Note. FI=frailty index; CI=confidence interval; BMI=body mass index.

[a]In FI × 100 units, which correspond percentage increases/decreases in FI levels at age 57 years (mean FI level at age 57 years was 0.161 among men and 0.174 among women).

[b]In percentage points per year from late midlife into old age (mean annual rate of change in FI levels from late midlife into old age was 0.28 percent/year among men and 0.34 percent/year among women). Point estimates are derived from models with the age × body composition interaction.

[c]Analysed individually. Linear mixed model adjusted with age, smoking, and adult socioeconomic status.

Supplementary Table 7. Categories of fat and lean mass index predicting point estimates of the FI level at age 57 years and the rate of change in FI levels from late midlife into old age assuming missing not at random sample attrition.

	**Level**[a]	**95 % CI**	**P**	**Rate of change**[b]	**95 % CI**	**P**
**Categories of fat and lean mass index**[c]						
High FMI & high LMI	6.96	5.13, 8.87	<0.001	0.004	-0.114, 0.121	0.970
High FMI & low LMI	4.83	2.65, 6.97	<0.001	0.002	-0.131, 0.143	0.964
Low FMI & low LMI	0.94	-1.014, 2.91	0.327	-0.021	-0.136, 0.095	0.765
Low FMI & high LMI	Ref.			Ref.		

Note. FI=frailty index; CI=confidence interval; FMI=fat mass index; LMI=lean mass index.

[a]In FI × 100 units, which correspond percentage increases/decreases in FI levels at age 57 years (mean FI level at age 57 years was 0.186).

[b]In percentage points per year from late midlife into old age (mean annual rate of change in FI levels from late midlife into old age was 0.34 percent/year). Point estimates are derived from models with the age × body composition interaction.

[c]Linear mixed model adjusted with age, smoking, and adult socioeconomic status.
